# Plant-Derived Cyclotides Modulate κ-Opioid Receptor Signaling

**DOI:** 10.1021/acs.jnatprod.1c00301

**Published:** 2021-07-26

**Authors:** Edin Muratspahić, Nataša Tomašević, Shahrooz Nasrollahi-Shirazi, Jasmin Gattringer, Fabiola Susanna Emser, Michael Freissmuth, Christian W. Gruber

**Affiliations:** † Center for Physiology and Pharmacology, Institute of Pharmacology, Medical University of Vienna, 1090 Vienna, Austria; ‡ Gaston H. Glock Research Laboratories for Exploratory Drug Development, Center for Physiology and Pharmacology, Medical University of Vienna, 1090 Vienna, Austria

## Abstract

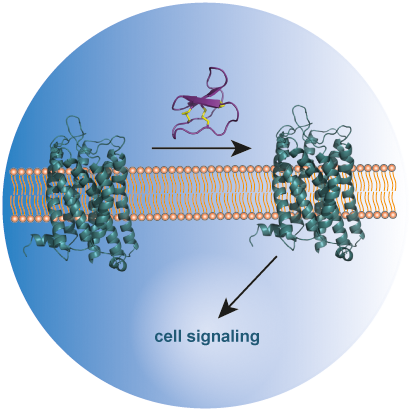

Cyclotides are plant-derived disulfide-rich peptides comprising a cyclic cystine knot, which confers remarkable stability against thermal, proteolytic, and chemical degradation. They represent an emerging class of G protein-coupled receptor (GPCR) ligands. In this study, utilizing a screening approach of plant extracts and pharmacological analysis we identified cyclotides from *Carapichea ipecacuanha* to be ligands of the κ-opioid receptor (KOR), an attractive target for developing analgesics with reduced side effects and therapeutics for multiple sclerosis (MS). This prompted us to verify whether [T20K]kalata B1, a cyclotide in clinical development for the treatment of MS, is able to modulate KOR signaling. T20K bound to and fully activated KOR in the low μM range. We then explored the ability of T20K to allosterically modulate KOR. Co-incubation of T20K with KOR ligands resulted in positive allosteric modulation in functional cAMP assays by altering either the efficacy of dynorphin A_1–13_ or the potency and efficacy of U50,488 (a selective KOR agonist), respectively. In addition, T20K increased the basal response upon cotreatment with U50,488. In the bioluminescence resonance energy transfer assay T20K negatively modulated the efficacy of U50,488. This study identifies cyclotides capable of modulating KOR and highlights the potential of plant-derived peptides as an opportunity to develop cyclotide-based KOR modulators.

Nature-derived disulfide-rich peptides are privileged molecules that have attracted increasing interest in drug discovery and development owing to their molecular architecture and promising pharmacological properties.^1^ Ziconotide and linaclotide are representative examples of disulfide-rich peptides that are available for clinical applications to treat severe chronic pain and irritable bowel syndrome with constipation, respectively.^2,3^ Another family of peptides with three disulfide bonds that provide great potential as scaffolds for peptide-based drug development and biopharmaceutical applications are cyclotides.^4^ These plant-derived molecules comprise around 30 amino acids composed of a cystine knot motif and a head-to-tail cyclic backbone.^5^ This unique topological fold endows cyclotides with exceptional thermostability and great resistance to chemical and enzymatic degradation.^6^ The diversity and abundance of cyclotides in nature is unprecedented: to date over 1200 sequences have been reported,^7^ and several transcriptome-mining/peptidomics approaches predict over 150 000 cyclotides to be discovered,^8−10^ with a single plant species capable of producing over 160 distinct cyclotides.^11^ The discovery of cyclotides in the early 1970s as uterotonic agents (i.e., stimulation of contractions of the uterus to accelerate childbirth) has prompted many studies reporting broad-spectrum bioactivities of cyclotides. While the native function of cyclotides is attributed to chemical defense of plants as they exhibit insecticidal^12−14^ and anthelmintic features,^15^ it is their pharmacological activities, such as anticancer,^16^ antibacterial,^17^ and immunosuppressive activities,^18,19^ that sparked interest for pharmaceutical applications.^20^

Recently, cyclotides have been reported as modulators of G protein-coupled receptors (GPCRs), today’s most druggable targets of the human genome.^21^ Deciphering molecular mechanisms underlying the uterotonic activity of an herbal remedy used in traditional African medicine has facilitated the identification of the cyclotide kalata B7 as a partial agonist of oxytocin and vasopressin V_1a_ receptors,^22^ typical class A GPCRs.^22^ A recent discovery of several cyclotides from *Carapichea ipecacuanha*, which antagonize the corticotropin releasing factor type I receptor,^23^ a class B GPCR, increased the diversity of GPCRs that can be modulated by cyclotides. Furthermore, taking advantage of an inherent biostability and structural diversity, cyclotides have been utilized as scaffolds for the design of stable and potent peptide ligands targeting other GPCRs.^24,25^

The κ-opioid receptor (KOR) together with the μ-OR (MOR), δ-OR (DOR), and nociceptin receptor (NOP) is important for regulation of nociception, reward and stress responses, and autonomic control.^26^ Recently, the KOR has been recognized as an alternative therapeutic target for the development of safer and effective analgesic drugs.^27^ In contrast to MOR agonists such as morphine and fentanyl, KOR agonists are not associated with addiction; however, their use is known to cause sedation, dysphoria, and hallucinations.^28^ In this context, biased KOR agonists that selectively activate the G protein pathway are promising candidates for the development of next-generation analgesics with improved side effect profiles to combat the ongoing opioid crisis.^27^ Additionally, several studies suggest a potential role of the KOR in multiple sclerosis (MS). Recently, Tangherlini et al. developed quinoxaline-based KOR agonists with anti-inflammatory and immunomodulatory activity in primary mouse and human immune cells as well as *in vivo* activity in an experimental autoimmune encephalomyelitis (EAE) mouse model of MS.^29^ Moreover, KOR ligands have been identified by high-throughput screening as remyelination-inducing compounds. Activating the KOR by agonists, such as U50,488 or nalfurafine, alleviates disease symptoms in the EAE model, by promoting oligodendrocyte differentiation and remyelination, as well as immune cell modulation.^30−32^ Thus, targeting the KOR represents an intriguing strategy to develop novel therapeutics for the treatment of pain and MS.^30−32^

Herein, we demonstrate a pharmacology-guided screening platform for the discovery of novel plant-derived peptide ligands of the KOR. Starting with peptide-enriched extracts of five plant species, we found cyclotides in ipecac root powder (*Carapichea ipecacuanha*) that bind to and activate the KOR. This triggered a detailed pharmacological characterization of the cyclotide [T20K]kalata B1, referred to as “T20K”, which is a drug candidate for the treatment of MS.^20^ We investigated whether T20K modulates KOR signaling, which may, at least in part, explain previously observed effects on reduced demyelination and reduced T-cell infiltration to the central nervous system (CNS) in the EAE model.^19^ Therefore, this study provides the first piece of evidence for utilizing plant-derived cyclotides to develop peptide-based KOR modulators for the treatment of pain and axonal degeneration.

## Results

### Screening of Disulfide-Rich Peptides for Binding to the KOR

**Figure 1:**
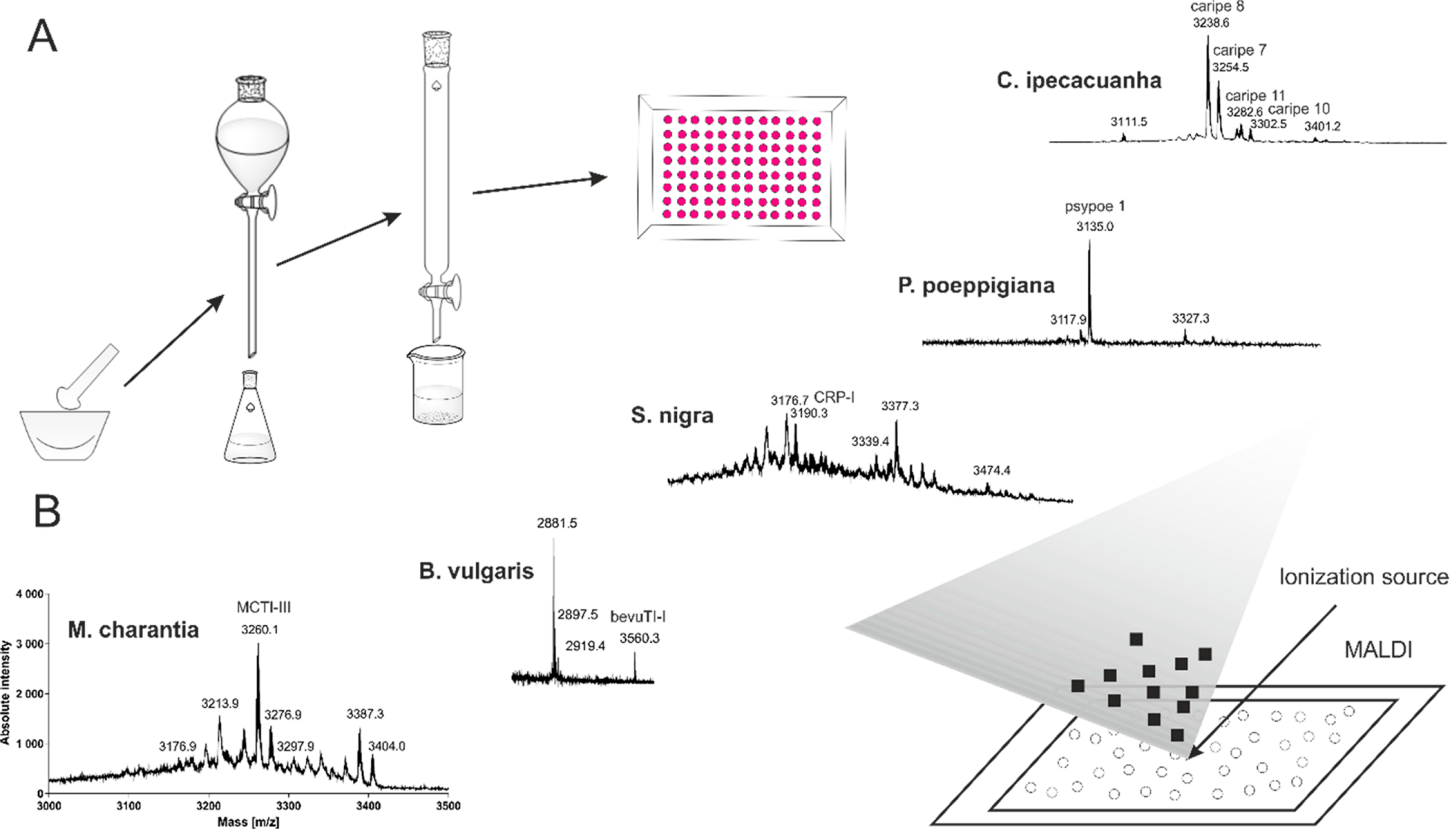
Screening approach of disulfide-rich plant extracts. (A) Flowchart of generation of disulfide-rich plant extracts. Powdered plant material was extracted with a mixture of dichloromethane/methanol (1:1, v/v) overnight followed by liquid/liquid-phase separation and C_18_ solid-phase extraction. A radioligand binding assay was used for initial pharmacological screening. (B) Respective mass spectra of disulfide-rich plant extracts acquired by MALDI-TOF/TOF mass spectrometry. Monoisotopic masses of MCTI-III (3260.1 Da), bevuTI-I (3560.3 Da), CRP-I (3190.3 Da), psypoe 1 (3135.0 Da), caripe 7 (3254.6 Da), caripe 8 (3238.5 Da), caripe 10 (3302.6 Da), and caripe 11 (3282.6 Da) are shown as [M + H]^+^. MALDI-TOF: matrix-assisted laser desorption ionization mass spectrometry-time-of-flight.

Driven by recent findings that cyclotides modulate GPCR signaling,^22,23^ we prepared extracts of plants that have previously been identified as a rich source of cyclotides including *C. ipecacuanha*(23) and *Psychotria poeppigiana*(33) (Figure 1). To increase the diversity of the plant extract library, we also included plant species known to contain other cyclotide-like or knottin peptides, such as *Momordica charantia*,^34^*Beta vulgaris*,^35^ and *Sambucus nigra* (Figure 1).^36^ Sequences of identified cysteine-rich peptides as well as information about their structural topology are provided in Table S1, Supporting Information.

**Figure 2:**
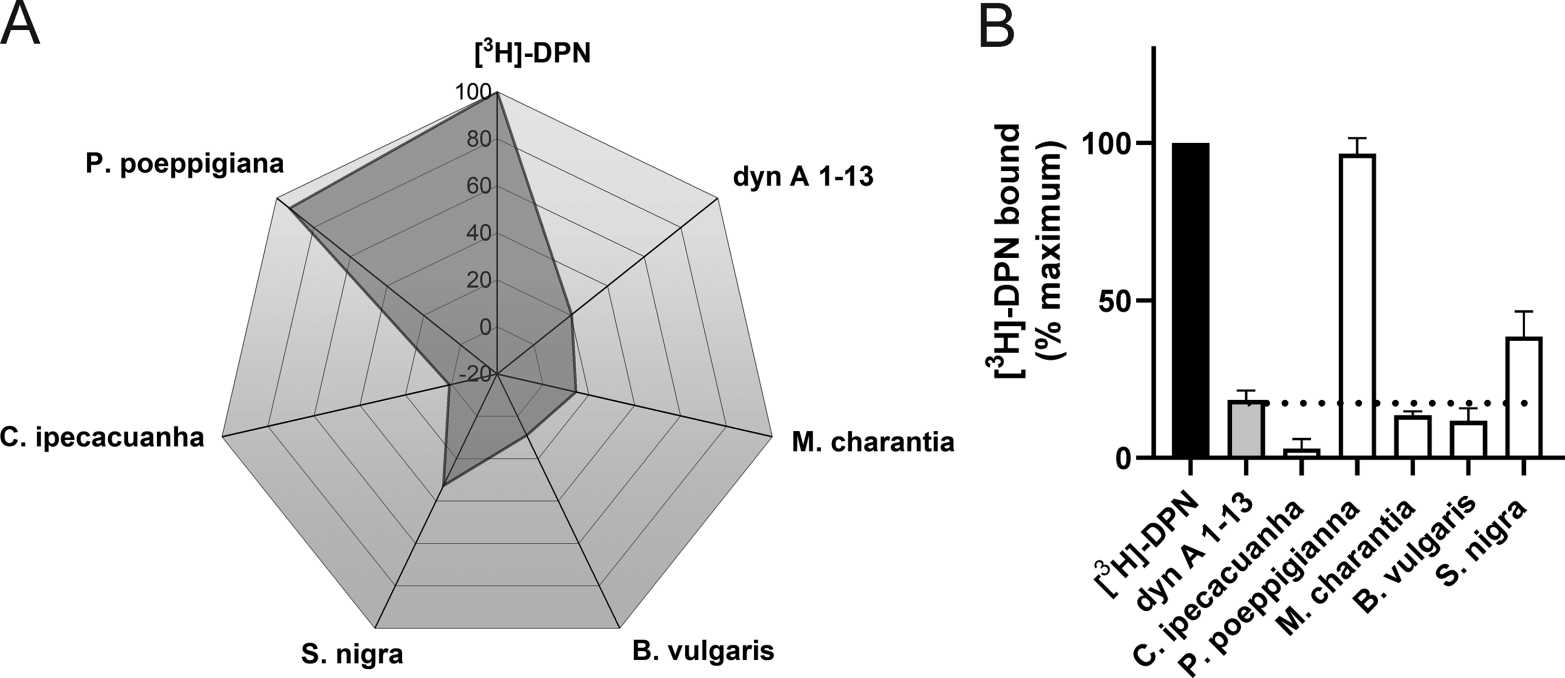
Binding effects of disulfide-rich plant extracts at the KOR. Data of (A) spider and (B) bar charts show the percentage of binding at concentrations of 300 μg/mL of plant extracts. Data are presented as mean ± SD (*n* = 2). Specific binding was obtained by subtraction of nonspecific from total binding (normalized to 100%), which is intended as binding of tritiated diprenorphine ([^3^H]-DPN, 1 nM, black bar) in the absence of competing ligand. Dynorphin (dyn) A_1–13_ (10 nM, light gray bar) was used as positive control. Extracts of *Momordica charantia*, *Beta vulgaris*, *Sambucus nigra*, *Carapichea ipecacuanha*, and *Psychotria poeppigiana* (white bars) were used for pharmacological studies.

Subsequently, we performed saturation binding experiment on HEK293 cell membrane preparations stably expressing mouse KOR to estimate the equilibrium dissociation constant (*K*_d_) of tritiated diprenorphine ([^3^H]-DPN) and the maximum number of receptor binding sites (*B*_max_) (Figure S1A, Supporting Information). *K*_d_ and *B*_max_ for the mouse KOR were 0.87 ± 0.06 nM and 7166 ± 147 femtomoles of ligand bound per milligram of membrane, respectively. These data were further used to determine the Hill slope of the saturation binding curve (1.00 ± 0.07), thus indicating that an incubation for 1 h at 37 °C is sufficient for [^3^H]-DPN to reach equilibrium in binding studies (Figure S1B, Supporting Information). After confirming the assay procedure, we studied the ability of peptide-enriched extracts to displace tritiated diprenorphine [^3^H]-DPN in a radioligand binding assay using membrane preparations from HEK293 cells stably expressing mouse KOR (Figure 2). Several plant extracts were able to displace the radioligand from the orthosteric binding pocket of the KOR, whereby the cyclotide-rich root extract of *C. ipecacuanha* exhibited the most pronounced binding effect (Figure 2).

### Cyclotides Isolated from Ipecac Are Ligands of the KOR

**Figure 3:**
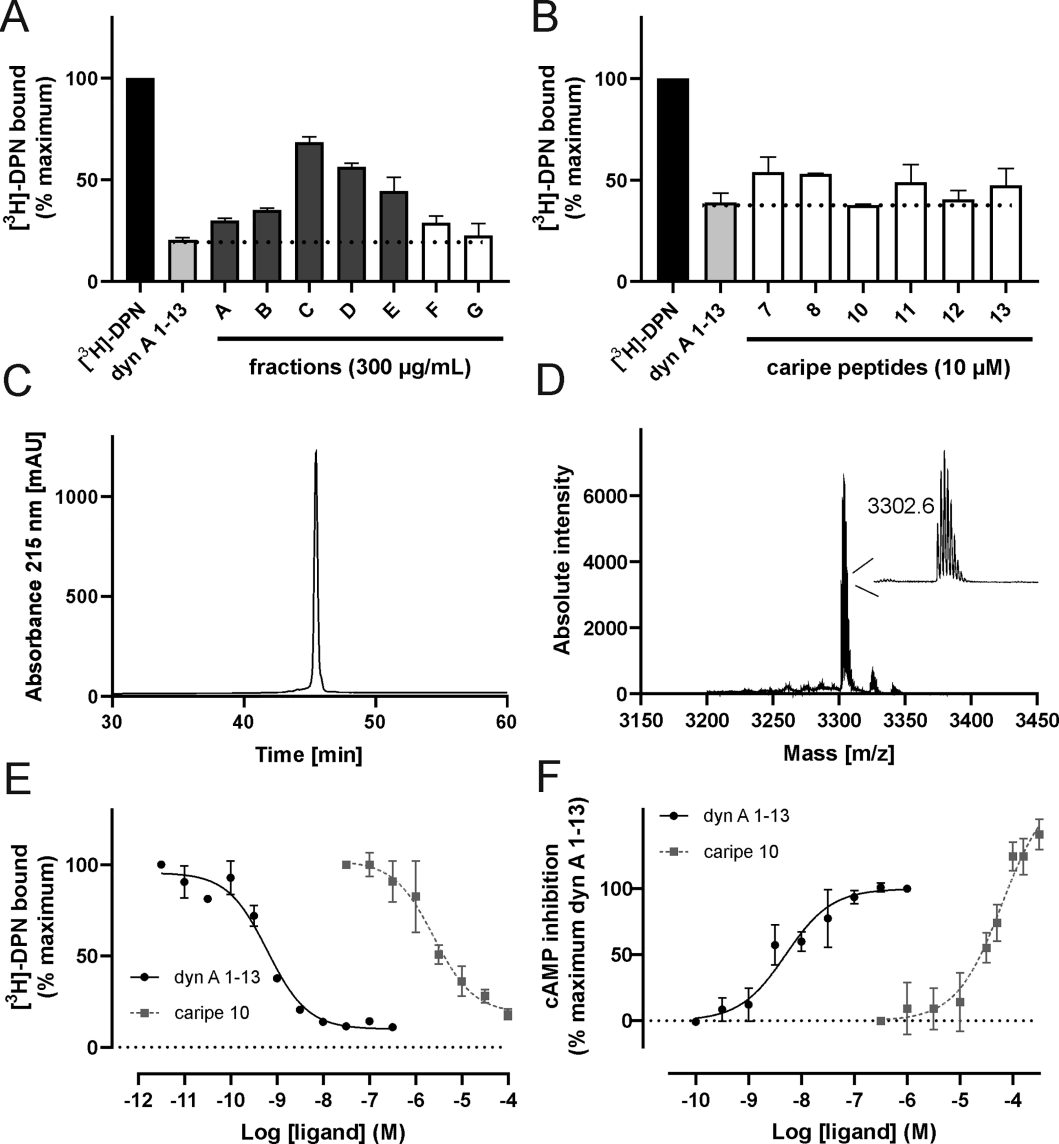
Receptor pharmacology of plant extracts and cyclotides from *C. ipecacuanha*. Binding data were obtained by measuring the displacement of [^3^H]-diprenorphine ([^3^H]-DPN, 1 nM, black bar) by (A) fractions with hydrophilic components (A–E, dark gray bars) and peptide-enriched fractions of *C. ipecacuanha* (F, G, white bars) and (B) cyclotides isolated from *C. ipecacuanha* (white bars). Dynorphin (dyn) A_1–13_ (10 nM, light gray bar) was used as control (*n* = 2). Quality control of caripe 10 analyzed by (C) RP-HPLC (purity of >95%) and (D) MALDI MS (inset, isotope peak pattern). (E) Concentration–response curves of caripe 10 (dark gray squares, *n* = 4) and dyn A_1–13_ (black circles, *n* = 2) in HEK293 cell membranes stably expressing the mouse KOR. Specific binding was obtained by subtraction of nonspecific binding from total binding. Data are mean ± SD and are normalized to the percentage of maximum binding. (F) Concentration-dependent cAMP inhibition following receptor activation by caripe 10 (dark gray squares, *n* = 3) and dyn A_1–13_ (black circles, *n* = 3) in HEK293 cells stably expressing the mouse KOR.

We next performed pharmacology-guided fractionation of the ipecac root extract (*C. ipecacuanha*) to identify cyclotides responsible for binding toward the KOR. Following chemical extraction and separation of cyclotide-rich fractions from early-eluting, hydrophilic compounds, we conducted radioligand binding experiments to test their affinity for the KOR. Fractions A–E, which mainly contain hydrophilic substances, displayed disparate displacement of [^3^H]-DPN at KOR (Figure 3A, Figure S2A–F, Supporting Information). Since emetine represents one of the most abundant alkaloids in ipecac root,^37^ we further measured the capability of emetine to displace [^3^H]-DPN at the KOR. Emetine did not displace the radioligand up to concentrations of 10 μM (Figure S3, Supporting Information). By contrast, fractions F and G were enriched with caripe cyclotides and exhibited strong ability to compete with the orthosteric radioligand (Figure 3A, Figure S2G and H, Supporting Information). While caripe 10 and 11 were the most abundant cyclotides in fraction F, fraction G contained high amounts of caripe 7, caripe 8, caripe 12, and caripe 13 (Figure S2G and H, Supporting Information). Therefore, we examined whether identified caripe cyclotides from the ipecac root,^23^ which belong to the bracelet family of cyclotides (Table S1, Supporting Information), are able to bind to the KOR. In fact, all six ipecac-derived cyclotides displaced the radioligand with varying efficiency of the KOR at a concentration of 10 μM (Figure 3B). Caripe 10 (molecular weight: 3302.6 Da) (Figure 3C and D), which had the strongest effect at 10 μM, was purified by RP-HPLC (purity >95%) and used for concentration-dependent binding studies and second messenger quantification at the KOR. Compared to dynorphin (dyn) A_1–13_, the endogenous peptide ligand of the KOR,^38^ caripe 10 displaced [^3^H]-DPN in a concentration-dependent manner with a *K*_i_ value of 1.2 ± 0.3 μM (Figure 3E). Since the KOR couples to the inhibitory α subunit of the heterotrimeric G protein and thus inhibits adenylyl cyclase activity, we assessed caripe 10 in a functional cAMP assay. Caripe 10 fully activated the KOR, associated with inhibition of cAMP production, with an *E*_max_ of 144 ± 12% and an EC_50_ of 60.2 ± 7.8 μM (Figure 3F).

### T20K Is a Full Agonist of the KOR

**Figure 4:**
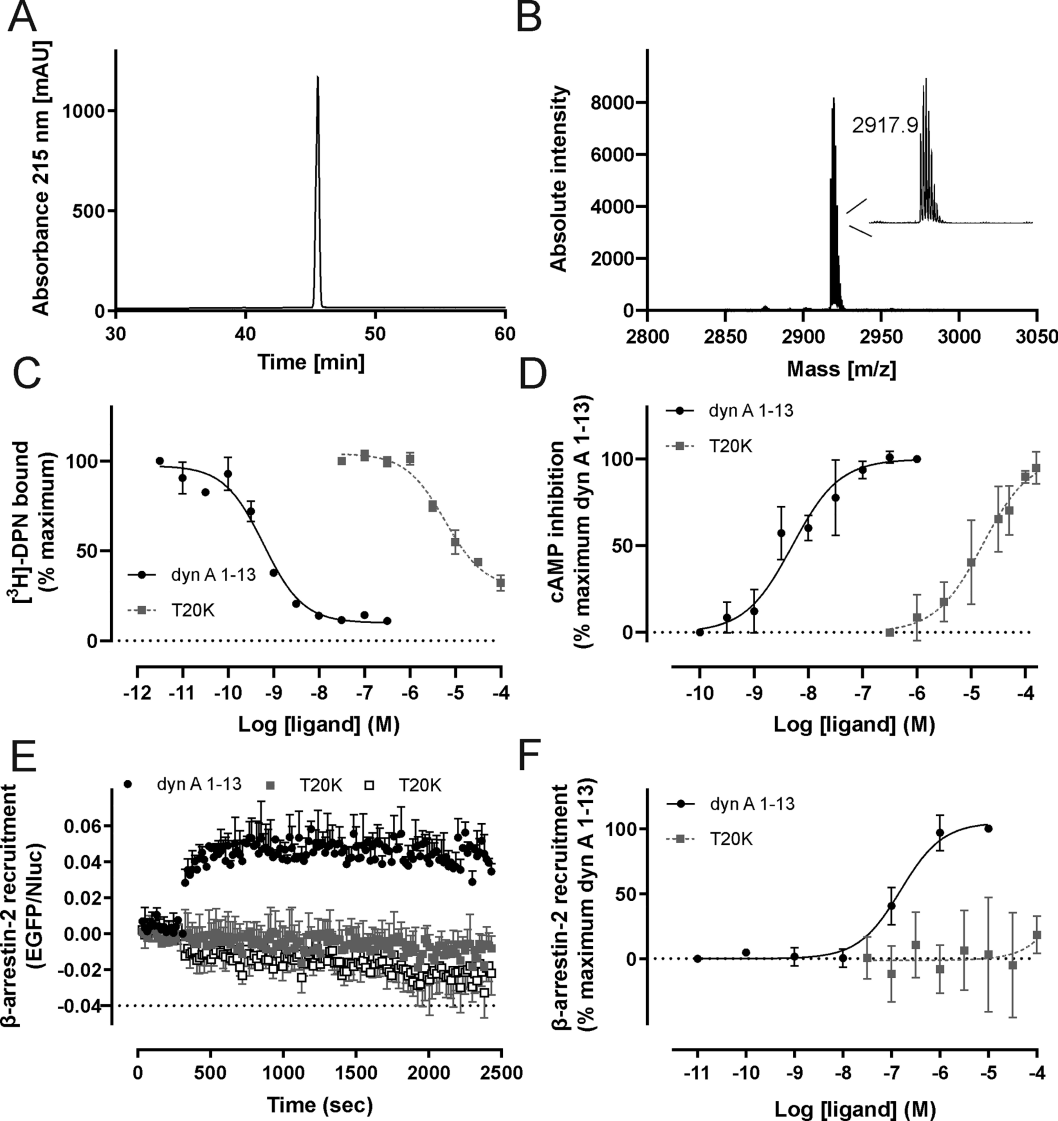
Radioligand binding and functional assays of T20K at the KOR. Purity (>95%) and molecular weight of T20K were characterized by (A) RP-HPLC and (B) MALDI MS (inset, isotope peak pattern). (C) Displacement of radioactive diprenorphine ([^3^H]-DPN, 1 nM) by T20K (dark gray squares, *n* = 3) and dyn A_1–13_ (black circles, *n* = 2) in HEK293 cell membranes stably expressing the KOR. Specific binding was obtained by subtraction of nonspecific from total binding. (D) Functional cAMP assay of T20K (dark gray squares, *n* = 4) and dyn A_1–13_ (black circles, *n* = 3) in HEK293 cells stably expressing the mouse KOR. (E) BRET kinetics were measured following coexpression of β-arrestin-2-nano luciferase (Nluc) and mouse KOR-EGFP and stimulation by 10 μM dyn A_1–13_ (black circles) and either 10 μM (dark gray squares) or 100 μM (white squares) T20K, 5 min after addition of the luciferase substrate (furimazine). The results are shown as differences in the BRET signals in the presence and absence of agonist and are expressed as the mean ± SD (*n* = 3). (F) Concentration response studies of T20K (dark gray squares, *n* = 3) and dyn A_1–13_ (black circles, *n* = 3) in a β-arrestin-2 recruitment assay conducted in HEK293 cells transiently expressing mouse KOR-EGFP and β-arrestin-2-Nluc and using furimazine as enzyme substrate. EGFP, enhanced green fluorescence protein; BRET, bioluminescence resonance energy transfer.

Our laboratory recently demonstrated that the synthetic cyclotide T20K, derived from kalata B1 (isolated from *Oldenlandia affinis*), a Möbius-type cyclotide (Table S1, Supporting Information), decreases clinical symptoms in the EAE model of MS following oral administration.^19^ Knowing that (i) caripe cyclotides activate KOR, (ii) T20K is a clinical candidate for the treatment for MS,^20^ and (iii) the KOR was identified as a therapeutic target in MS models,^29−31^ we interrogated whether T20K could modulate KOR signaling as well. Therefore, we obtained T20K (molecular weight: 2917.9 Da, purity >95%) (Figure 4A and B) for pharmacological characterization. We first conducted a time course experiment to verify that an incubation of 1 h at 37 °C, as used for caripe 10, is sufficient to reach equilibrium. In fact, compared to 1 nM [^3^H]-DPN, which is in equilibrium already after 30 min, an incubation of 60 min was sufficient for T20K to reach equilibrium in the presence of 1 nM [^3^H]-DPN (Figure S4, Supporting Information). Our binding and functional data indicate that T20K concentration-dependently displaces [^3^H]-DPN from the KOR binding site with a *K*_i_ of 2.0 ± 0.5 μM and fully activates the KOR with an *E*_max_ of 105 ± 5% and EC_50_ of 24.0 ± 13.5 μM (Figure 4C and D). Since β-arrestins may be involved in the development of KOR side effects including dysphoria and sedation,^28^ we measured the ability of T20K to recruit β-arrestin-2 at the KOR in a bioluminescence resonance energy transfer (BRET)-based assay. Consequently, upon incubation of T20K with HEK293 cells transiently coexpressing β-arrestin-2-nano luciferase (Nluc) and EGFP-KOR, no interaction of β-arrestin-2 and KOR was detected up to concentrations of 100 μM (Figure 4E and F). By contrast, dyn A_1–13_ recruited β-arrestin-2 with an EC_50_ of 159.0 ± 92.4 nM. These data suggest that T20K is a full agonist of the KOR and as compared to dyn A_1–13_ has a reduced potency to recruit β-arrestin-2.

### T20K Is an Allosteric Modulator of the KOR

In addition to the orthosteric site where cognate ligands bind, GPCRs comprise additional allosteric sites that fine-tune their activity profile.^39^ Recently, Che and colleagues identified allosteric nanobody-based modulators of the KOR.^40^ Accordingly, using radioligand binding and functional assays we examined any allosteric effects of T20K at the KOR. Radioligand binding studies were conducted in HEK293 cells stably expressing the mouse KOR and co-incubating varying concentrations of T20K with either dyn A_1–13_ or U50,488, a selective KOR agonist. We demonstrated that T20K displaces [^3^H]-DPN from the orthosteric binding site, but only slightly affects the affinity of endogenous dyn A_1–13_ (Figure 4A, Table 1). To analyze a probe-dependent effect, we co-incubated T20K with U50,488 and observed no apparent change in the affinity of U50,488 (Figure 4B, Table 2). At the functional level, by measuring cellular cAMP levels, we demonstrated that T20K does not affect the potency of dyn A_1–13_, but instead leads to a significant increase in its efficacy by ∼30–60% when using 0.3, 1, and 3 μM T20K (Figure 4C, Table 1). These effects were KOR-dependent, as no changes in cAMP levels were observed for dyn A_1–13_ alone or in the presence of 10 μM T20K in untransfected HEK 293 cells (Figure S5, Supporting Information). On the other hand, co-incubation with U50,488 significantly increased basal cAMP production by ∼50% and enhanced potency of U50,488, yet only with 10 μM T20K. Furthermore, the maximal U50,488-induced cAMP response increased to approximately 120% when co-incubated with 10 μM T20K (Figure 4D, Table 2). We further co-incubated T20K with U50,488 to explore if T20K has an impact on the ability of U50,488 to recruit β-arrestin-2. Co-treatment of the KOR with 10 μM T20K slightly, yet significantly, alleviated efficacy of the U50,488 by ∼25%, with no apparent changes in its potency (Figure 4E and F, Table 2). These data suggest that T20K can modulate KOR signaling.

**Figure 5:**
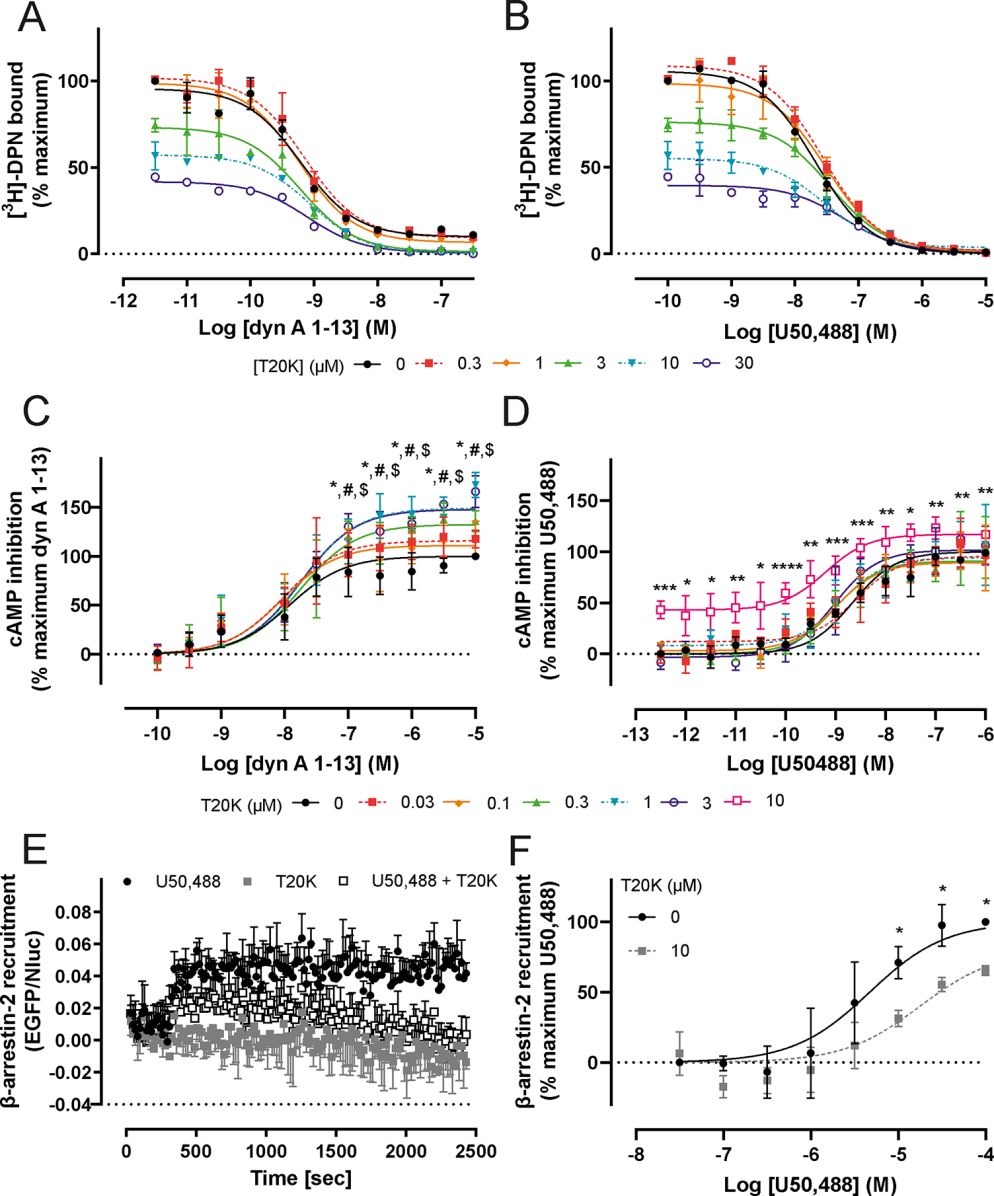
Allosteric effects of T20K at the KOR. Displacement radioligand binding of [^3^H]-diprenorphine ([^3^H]-DPN, 1 nM) by co-incubation of 0.3 μM (red squares), 1 μM (orange diamonds), 3 μM (light green triangles), 10 μM (dark green inverted triangles), and 30 μM (open blue circles) concentrations of T20K with distinct concentrations of (A) dyn A_1–13_ (black circles) or (B) U50,488 (black circles) in HEK293 cell membranes stably expressing the mouse KOR. Data are shown as mean ± SD of specific binding, which was obtained by subtraction of nonspecific from total binding (*n* = 2). Functional cAMP assay of 0.03 μM (red squares), 0.1 μM (orange diamonds), 0.3 μM (light green triangles), 1 μM (dark green inverted triangles), 3 μM (open blue circles), and 10 μM (open pink squares) T20K in combination with (C) dyn A_1–13_ (black circles) or (D) U50,488 (black circles) in HEK293 cells stably expressing the KOR. Data are normalized to percentage of maximal activation, detected at the highest endogenous/orthosteric ligand concentration, and are shown as mean ± SD (*n* = 4 or *n* = 7 for 10 μM T20K). Statistical significance was determined by two-way ANOVA followed by Dunnett’s test. Asterisks denote significance between dyn A_1–13_ alone and 3 μM T20K (*p < 0.05) or U50,488 alone and 10 μM T20K (*p < 0.05; **p < 0.01; **p < 0.001; ****p < 0.0001). Hashtag and dollar symbols indicate significance between dyn A_1–13_ alone vs co-incubation with 1 μM or 0.3 μM T20K (^*#*^*p <*0.05; ^$^*p <*0.05). (E) BRET kinetics measurements (*n* = 3) were conducted with U50,488 (black circles, 10 μM), T20K (dark gray squares, 10 μM), or a combination of both (white squares, 10 μM each) in HEK293 cells after transient coexpression of mouse KOR-EGFP and β-arrestin-2-nano luciferase (Nluc). (F) Concentration response curves of T20K (dark gray squares) in combination with U50,488 (black circles) in the β-arrestin-2 recruitment assay generated using HEK293 cells transiently expressing mouse KOR-EGFP and β-arrestin-2-Nluc. The results are presented as differences in the BRET signals in the presence and absence of ligands and are expressed as the mean ± SD (*n* = 4). Asterisks represent significance between U50,488 alone and 10 μM T20K (*p < 0.05). EGFP, enhanced green florescence protein; BRET, bioluminescence resonance energy transfer.

**Table 1:** Pharmacological Data of dyn A_1-13_ with or without T20K at the KOR^a^

ligand co-incubation	affinity	potency/efficacy cAMP inhibition	
dyn A_1–13_ ± T20K (μM)	*K*_i_ ± SD (nM)	EC_50_ ± SD (nM)	*E*_max_ ± SD (%)
0	0.3 ± 0.1	6.7 ± 0.9	100
0.03	n.d.	6.8 ± 1.1	115.5 ± 20.0
0.1	n.d.	5.0 ± 0.4	122.0 ± 21.6
0.3	0.4 ± 0.1	8.8 ± 7.0	133.2 ± 41.9*
1	0.2 ± 0. 1	8.8 ± 2.4	160.3 ± 47.4**
3	0.3 ± 0.1	16.5 ± 7.2	158.8 ± 54.0*
10	0.4 ± 0.1	n.d.	n.d.
30	0.4 ± 0.1	n.d.	n.d.

a Data are from two to four independent biological replicates. Asterisks indicate significance between *E*_max_ values of dyn A_1–13_ alone and a combination of dyn A_1–13_ with either 0.3, 1, or 3 μM T20K. Student’s *t* test (*p < 0.05; **p < 0.01). n.d., not determined; dyn, dynorphin.

**Table 2:** Pharmacological Data of U50,488 with or without T20K at the KOR^a^

ligand co-incubation	affinity	potency/efficacy cAMP inhibition		potency/efficacy arrestin recruitment	
U50,488 ± T20K (μM)	*K*_i_ ± SD (nM)	EC_50_ ± SD (nM)	*E*_max_ ± SD (%)	EC_50_ ± SD (nM)	*E*_max_ ± SD (%)
0	8.1 ± 1.1	1.7 ± 0.8	100	4886 ± 3082	100
0.03	n.d.	0.8 ± 0.1	91.1 ± 6.5	n.d.	n.d.
0.1	n.d.	0.9 ± 0.4	90.0 ± 8.4	n.d.	n.d.
0.3	10.2 ± 0.4	0.9 ± 0.3	88.2 ± 18.7	n.d.	n.d.
1	15.3 ± 0.8	0.9 ± 0.2	92.0 ± 12.5	n.d.	n.d.
3	17.2 ± 1.2	1.0 ± 0.1	99.9. ± 11.8	n.d.	n.d.
10	15.2 ± 0.6	0.5 ± 0.2*	121.5 ± 8.3***	10 411 ± 7672	77.3 ± 9.4**
30	35.9 ± 8.1	n.d.	n.d.	n.d.	n.d.

a Data are from two to seven independent biological replicates. Asterisks indicate significance between EC_50_ or *E*_max_ values of U50,488 alone and a combination of U50,488 with 10 μM T20K. Student’s *t* test (*p < 0.05; **p < 0.01). n.d., not determined.

## Discussion

An unmet clinical need for safer and effective analgesics and the opioid crisis have unlocked the therapeutic potential of the KOR.^28^ Difelikefalin, a peripherally acting tetrapeptide KOR agonist, has recently been approved to treat postoperative pain.^41^ Further, G protein-biased KOR ligands hold promise to develop next-generation analgesics with fewer central dysphoric- and sedative-like side effects for the treatment of chronic pain.^42,43^ The recent discovery that KOR agonism mediates beneficial effects in the EAE mouse model augments its therapeutic potential to develop novel MS therapies.

Encouraged by (i) cyclotides’ ability to modulate GPCR signaling^24,25^ and (ii) the promising therapeutic potential of the KOR, in the present study we aimed at discovering cyclotide ligands that target the KOR. Creating and screening libraries of disulfide-rich peptide-based plant extracts in radioligand binding studies integrated with a bioactivity-guided fractionation approach enabled us to identify several cyclotides from *C. ipecacuanha* that bind to the KOR. One of these peptides, caripe 10, showed the ability to displace [^3^H]-DPN in the low μM range, while it fully activated KOR with an EC_50_ of ∼60 μM in the functional cAMP assay. Interestingly, our previous study identified caripe 8 as an antagonist of the corticotropin releasing factor type I receptor.^23^ The observation that caripe peptides modulate KOR signaling motivated us to explore whether a clinically relevant analogue of native kalata B1, that is, T20K, is able to act as a ligand of the KOR. Similar to caripe 10, T20K bound to the KOR in the low μM range. Our functional data further demonstrated that T20K is a full agonist of the KOR with an EC_50_ of ∼24 μM. The moderate affinity and potency of caripe 10 and T20K are in line with kalata B7, a ligand of oxytocin and vasopressin V_1a_ receptors:^22^ the larger size of cyclotides may hinder deep penetration of the receptor binding pocket.^44^

Allosteric modulation of GPCRs has emerged as an attractive approach for developing potential therapeutics for the treatment of CNS disorders.^45^ Allosteric modulators of GPCRs bind to less highly conserved, topologically distinct sites from the orthosteric sites, allowing for greater receptor subtype selectivity.^45^ Using high-throughput screening Burford et al. discovered positive and silent allosteric modulators of MOR in a β-arrestin recruitment assay.^46^ Recently, leveraging X-ray crystallography Che and colleagues identified a nanobody-targeted allosteric binding site at the KOR.^40^ Hence, this prompted us to explore a possible allosteric mode of T20K at the KOR in combination with the endogenous ligand dyn A_1–13_ and the synthetic selective agonist U50,488 in radioligand binding studies and functional assays. Whereas no apparent changes in the affinity of dyn A_1–13_ were observed, costimulation with T20K increased the efficacy of the endogenous peptide ligand. To further verify allosteric effects of T20K at the KOR, we co-incubated it with U50,488. Herein, we monitored probe dependence: while there was a negligible increase in the affinity of U50,488, cell cotreatment with T20K led to a minor increase in the efficacy and potency of U50,488, albeit only at the highest concentration of 10 μM. This effect was accompanied by an increase in the basal activity following cell treatment with 10 μM T20K. On the other hand, T20K altered the ability of U50,488 to induce β-arrestin-2 recruitment by moderately decreasing its efficacy. Receptor binding studies demonstrated that T20K displaces [^3^H]-DPN, suggesting that T20K interacts with the KOR at the orthosteric binding site. Accordingly, whether a KOR binding pocket has sufficient capacity to accommodate both T20K and an agonist simultaneously warrants further investigations. However, KOR is known to form homodimers in recombinant cell systems.^47^ In such a scenario with two binding pockets of the KOR homodimer T20K and dyn A_1–13_ or U50,488 may be able to occupy each binding pocket at the same time, thereby enabling T20K to exert its allosteric action. This hypothesis is only valid when using low concentrations of T20K since T20K at higher concentrations will most likely occupy both binding pockets. A similar mode of action has been recently proposed for ignavine, an allosteric modulator of the MOR, which is an active ingredient of *Aconiti* tubers.^48^ Furthermore, structural studies of GPCRs bound to peptide allosteric modulators provided mechanistic insights into how peptides allosterically regulate GPCRs. For instance, Maeda and colleagues have recently demonstrated that the allosteric modulator muscarinic toxin MT7 interacts with extracellular loop 2 and the extracellular end of transmembrane 6 of muscarinic acetylcholine receptor M1, thereby explaining its high receptor subtype selectivity.^49^ Thus, based on this structural information it is possible that T20K interacts with extracellular loop 2 of KOR to exhibit its allosteric mode of action. In fact, simulation studies have uncovered direct involvement of extracellular loop 2 in binding affinity and activation of KOR by dyn A.^50−52^

We have previously demonstrated that T20K inhibits human T-cell proliferation by an IL-2-dependent mechanism.^53^ In a follow-up study, the *ex vivo* treatment of immune cells derived from the spleen of EAE mice with T20K resulted in a concentration-dependent downregulation of T-cells and certain cytokines.^19^ Furthermore, using immunohistochemistry we observed no significant infiltration of mononuclear cells and an intact myelin sheath of the spinal cord following T20K treatment *in vivo*.^19^ This led to a significant reduction of inflammation and a lower grade of demyelination in the CNS.^19^ Recent studies revealed that quinoxaline-based small molecules reduced expression of pro-inflammatory cytokines in human and mouse immune cells in a KOR-dependent fashion.^29^ These effects have been further corroborated in the EAE model where the compound decreased disease severity by downregulating effector T cell activation.^29^ Moreover, KOR agonism has been identified as a strategy to promote oligodendrocyte differentiation and remyelination.^30,31^ Du et al. demonstrated that administration of U50,488 significantly reduces disease scores in EAE as well as demyelination and leukocyte infiltration into the spinal cord.^31^ U50,488 also induced remyelination in the cuprizone- and lysolecithin-based demyelination mice models.^30,31^ KOR agonism has been further emphasized by Mei et al., who demonstrated an increased differentiation of human oligodendrocyte progenitor cells into mature oligodendrocytes upon U50,488 treatment.^30^ However, the therapeutic potential of U50,488 is limited, as it causes deleterious side effects including dysphoria and aversion.^54,55^ In fact, biased signaling at the KOR represents a therapeutic strategy that allows minimizing adverse effects while favoring optimum therapeutic efficacy. Nalfurafine is a KOR agonist clinically approved in Japan for the treatment of uremic pruritis, which is biased toward G protein pathways.^56^ Denny and colleagues reported more effective disease reduction in EAE mice by nalfurafine than U50,488, highlighting its potential to promote recovery and remyelination and thus clinical use in MS.^32^ Hence, these studies allow us to speculate that previously reported reduced T-cell infiltration and demyelination of T20K^19^ may, at least in part, be explained by its modulation of the KOR. In fact, the observed affinity (∼2 μM) and potency (∼24 μM) values of T20K at the KOR are in line with T20K’s potency (∼2–4 μM) to suppress lymphocyte proliferation.^53^

Plant-derived cyclotides continue to be an exciting source of inspiration for biopharmaceutical applications. With a molecular weight of ∼3000 Da they bridge the gap between small molecules (<500 Da) and large biologics (>5000 Da).^24^ Their topologically stable structure in combination with amenability to combinatorial sequence variations further warrants the potential of cyclotides in the design and development of peptide-based therapeutics.^57^ In this study, we identified cyclotides as modulators of the KOR, an emerging target for developing therapeutics for pain and MS. Modulation of KOR signaling by cyclotides provides evidence that cyclotides may be exploited as templates to design orthosteric cyclotide KOR ligands or allosteric cyclotide-based KOR modulators with improved pharmacological profiles. In that respect, the molecular grafting approach has been successfully employed to design potent cyclotide-based agonists and antagonists targeting the melanocortin 4 receptor,^58^ the bradykinin B_1_ receptor,^59^ and the C-X-C chemokine receptor type 4.^60^ Herein, we not only expand the repertoire of GPCRs that cyclotides target but also highlight their potential to design novel cyclotide-based allosteric modulators of GPCRs. Plants have historically been a rich source of therapeutic agents, from widely used analgesics such as morphine to antimalarial medicines including artemisinin.^61^ At the very least, our study reinforces the great potential of plant-derived cyclotides for drug discovery.

## Experimental Section

### Materials

Dyn A_1–13_ amide trifluoroacetate salt was purchased from Bachem (Austria). Emetine hydrochloride and naloxone were obtained from Sigma (Austria). [^3^H]-Diprenorphine ([^3^H]-DPN) was from PerkinElmer (Austria), and the cAMP G_i_ kit from Cisbio (Germany). jetPRIME transfection reagent was from Polyplus (Austria). T20K was obtained as a kind gift from Cyxone AB.

### Plant Extraction

The extracts of *C. ipecacuanha*, *M. charantia*, *B. vulgaris*, *S. nigra*, *P. poeppigiana*, and *C. ipecacuanha* (Alfred Galke GmbH, Germany) and peptide-enriched fractions have been prepared as previously described.^22^ Briefly, the dried plant material (50 g) was extracted with 1 L of methanol/dichloromethane, 1:1 (v/v), overnight under continuous agitation at room temperature. After removing plant material and filtration a 0.5 volume of ddH_2_O was added to the extract, and the methanol/water phase containing peptides of interest was separated from the organic phase. The aqueous phase was then evaporated, lyophilized, and then subjected to C_18_ solid-phase extraction. The dried, crude extract was dissolved in 10% methanol/90% ddH_2_O (v/v) and loaded onto the C_18_ material ZEOprep 60 Å, irregular 40–64 μm (Zeochem, Uetikon, Switzerland). After equilibration of the column with solvent A (99.9% ddH_2_O/0.1% trifluoroacetic acid, v/v) and washing with 10–30% solvent B (90% acetonitrile/9.92% ddH_2_O/0.08% trifluoroacetic acid, v/v/v) it was eluted with 50–80% solvent B, depending on the plant extract, to separate the peptide-containing fractions from hydrophilic components. The mass of peptide-enriched fractions was monitored by MALDI mass spectrometry.

### Peptide Analysis with MALDI Mass Spectrometry

Analysis of peptide-enriched fractions was conducted by a MALDI-TOF/TOF 4800 analyzer (AB Sciex, Framingham, MA, USA) in a reflector positive ion mode acquiring 2000 to 10 000 total shots per spectrum with a laser intensity of 3500. Sample preparation was carried out using an α-cyano-hydroxyl-cinnamic acid (α-CHCA) matrix (Sigma–Aldrich, St. Louis, MO, USA) dissolved in ddH_2_O/acetonitrile/trifluoroacetic acid, 50/49.9/0.1% (v/v/v) (final concentration 5 mg/mL). A 0.5 μL amount of each sample was mixed with 3 μL of matrix solution and spotted directly onto the MALDI target plate. Spectra were acquired, processed, and analyzed using the Data Explorer Software (AB Sciex).

### Bioactivity-Guided Fractionation of Cyclotides

The bioactivity-guided fractionation of the cyclotide-enriched ipecac extract was performed as previously described.^23^ The extract dissolved in 5% buffer B was manually loaded onto the preparative Phenomenex Jupiter C_18_ column (250 mm × 21.2 mm, 10 μm, 300 Å; Phenomenex, Aschaffenburg, Germany). The mobile phase was composed of solvent A (99.9% ddH_2_O/0.1% trifluoroacetic acid, v/v) and solvent B (90% acetonitrile/9.92% ddH_2_O/0.08% trifluoroacetic acid, v/v/v). The automatic fractionation was performed on a Dionex 3000 LC unit (Dionex, Amsterdam, The Netherlands) machine using a linear gradient of solvent B between 5% and 65% at a flow rate of 8 mL/min. Analytical RP-HPLC was conducted on a Kromasil C_18_ column (250 mm × 4.6 mm, 5 μm, 100 Å; dichrom GmbH, Marl, Germany) using a linear gradient of solvent B between 5% and 65% at a flow rate of 1 mL/min. The elution of peptides was monitored via UV absorbance at 214, 254, and 280 nm wavelengths.

### Cell Culture, Transfection, and Cloning

HEK293 cells were cultured in Dulbecco’s modified Eagle’s medium (DMEM) containing 10% fetal bovine serum and 50 U/mL penicillin and streptomycin and were grown at 37 °C and 5% CO_2_. Cell transfection using 2 μg (six-well plates) or 10 μg (10 cm plates) of plasmids expressing mouse KOR tagged with EGFP and human β-arrestin-2 fused to Nluc was performed with jetPRIME transfection reagent according to the manufacturer’s protocol (Polyplus). Mouse KOR was introduced N-terminally into the pEGFP-N1 vector with *Bam*HI and *Hin*dIII restriction sites. The HEK293 cell line stably expressing mouse KOR-EGFP was produced by using the selection marker geneticin disulfate (0.8 mg/mL of G418, ROTH, Austria) and flow cytometry for the identification of cells expressing mouse KOR-EGFP. Radioligand competition binding studies were employed to identify positive clones.

### Radioligand Competition Binding Assays

Membranes were prepared using a stable mouse KOR HEK293 cell line as previously described.^62^ A radioligand binding assay was performed in duplicates and standard binding buffer containing 50 mM Tris-HCl (pH 7.4), 10 mM MgCl_2_, and 0.1% bovine serum albumin. A saturation binding assay of [^3^H]-DPN was carried out in standard binding buffer on HEK293 cells stably expressing mouse KOR. For competition binding, 75 μL each of [^3^H]-DPN (1 nM final), peptides (4×), and membranes (5–7 μg/assay) were incubated in the standard binding buffer, and both saturation and competition binding mixtures were incubated for 1 h at 37 °C. A final concentration of 10 μM naloxone was used for nonspecific binding for both saturation and competition binding studies. The allosteric effects of T20K were measured by co-incubating 75 μL of each of [^3^H]-DPN (1 nM final), T20K (4×), dyn A_1–13_, or U504,88 (4×) and membrane preparations (5–7 μg/assay). Time course analysis was performed by incubating 75 μL of 1 nM (final) [^3^H]-DPN either alone or with 75 μL of 10 μM T20K (4×) in the binding buffer over 90 min at 37 °C. Bound and free ligand was separated by rapid filtration using a 0.1% polyethlyenimine-soaked GF/C glass fiber filter and a Skatron cell harvester.

### cAMP Assay

The quantification of cAMP levels was carried out in triplicates with either HEK293 cells stably expressing mouse KOR or untransfected cells using the homogeneous time-resolved fluorescence resonance energy transfer (HTRF) cAMP-Gi kit (CisBio, Codolet, France) as per the manufacturer’s protocol with minor changes. Briefly, 2000 cells per 5 μL per well were seeded into a white 384-well plate and incubated with 5 μL of indicated concentrations of peptide solutions prepared (2×) in 1× stimulation buffer (supplemented with IBMX; 0.5 mM final) and forskolin (10 μM final). The reaction mixture was incubated at 37 °C for 30 min. To measure the allosteric modulation, the cells were pretreated with T20K (4×) for 30 min at 37 °C followed by co-incubation of dyn A_1–13_ or U50,488 (4×) and forskolin (10 μM final) for an additional 30 min at 37 °C. After the addition of europium cryptate-labeled cAMP and cAMP d2-labeled antibody (5 μL of each) and an incubation for 1 h at room temperature, cAMP quantification was measured by HTRF on a Flexstation 3 (Molecular Devices, San Jose, CA, USA) using the ratio 665/620 nm.

### β*-*Arrestin-2 Recruitment Assay

The β-arrestin-2 recruitment assay was performed on HEK293 cells transiently coexpressing human β-arrestin-2-Nluc and mouse KOR-EGFP in a 1:10 ratio. At 16–24 h post-transfection, cells were transferred into white clear-bottom cell culture plates in phenol red-free DMEM supplemented with 10% fetal bovine serum (FBS) at a density of 50 000 cells/100 μL/well and incubated overnight at 37 °C. Subsequently, the cells were serum starved for 1 h at 37 °C in phenol red-free DMEM. Furimazine (Promega, Madison, WI, USA), diluted 1:50, and peptide concentrations were prepared (4×) in Hank’s balanced salt solution (HBSS), and the assay was performed in triplicates. A 50 μL amount of furimazine was added to the cells and incubated for 5 min at 37 °C. After establishing the baseline for 5 min, ligands were added, and the response was measured for 35 min (BRET kinetics). Concentration–response curves were generated by the BRET signal measured following incubation of various ligand concentrations for 5 min at 37 °C. The allosteric modulation of T20K and U50,488 at KOR was measured by co-incubation. Filtered light emissions were sequentially measured at 460 nm for Nluc and 510 nm for EGFP using the Flexstation 3.

### Data Analysis

Data analysis was performed using GraphPad Prism (GraphPad Software, San Diego, CA, USA). Concentration–response curves of functional assays were generated by fitting the data to three-parameter nonlinear regression curves with a bottom and top constrained to 0 and 100, respectively, and a slope of 1 to obtain potency (EC_50_) and maximum efficacy (*E*_max_). Concentration–response curves of functional assays for measuring allosteric modulation were produced by fitting the data to three-parameter nonlinear regression curves without constraints and a slope of 1 except for dyn A_1–13_ or U50,488, which were constrained to 0 (bottom) and 100 (top). Graphs were normalized to 100%, which corresponds to the highest concentration of the positive control, which is either dyn A_1–13_ or U50,488 used in the assay. IC_50_ and inhibition constant (*K*_i_) values from radioligand competition binding assays were calculated by fitting the data to a three-parameter logistic Hill equation and using the Cheng and Prusoff approximation. *K*_d_ and *B*_max_ values were calculated by fitting saturation binding data to a one-site binding model, whereas the Hill slope was assessed using the four-parameter logistic Hill equation. Data from competition binding studies were normalized to maximum percentage (100%) of specific binding of [^3^H]-DPN, which refers to an average of 4000–5000 fmol/mg protein for KOR. Statistical significance was calculated using Student’s *t* test or two-way ANOVA followed by Dunnett’s test.

## Supporting Information Available

Radioligand binding data of [3H]-DPN, T20K, and emetine; functional cAMP data of dyn A_1-13_ in the absence and presence of T20K; HPLC fractionation of ipecac extract; sequences and structural details of cysteine-rich peptides identified in plant extracts (PDF)
