# Supplementary material for: Plant-Derived Cyclotides Modulate κ-Opioid Receptor Signaling
Source: J Nat Prod. 2021 Jul 26;84(8):2238–48. doi: 10.1021/acs.jnatprod.1c00301 (PMC8406418; doi:10.1021/acs.jnatprod.1c00301)
Supplement: Supplementary file 1 — np1c00301_si_001.pdf [file np1c00301_si_001.pdf]

Supporting Information

Plant-derived cyclotides modulate κ-opioid receptor signaling

Edin Muratspahić, Nataša Tomašević, Shahrooz Nasrollahi-Shirazi, Jasmin Gattringer, Fabiola Susanna Emser, Michael Freissmuth, Christian W. Gruber

List of Supporting Information

Supporting Information Figure S1. Saturation binding of [^{3}H]-DPN at the mouse KOR.

Supporting Information Figure S2. HPLC fractionation of ipecac extract.

Supporting Information Figure S3. Receptor pharmacology of emetine at the KOR.

Supporting Information Figure S4. Time course analysis of [^{3}H]-DPN and T20K in the radioligand binding assay at the KOR.

Supporting Information Figure S5. cAMP assay of dyn A_{1-13} without or with T20K in un-transfected HEK293 cells.

Supporting Information Table S1. Identification of cysteine-rich peptides plant extracts.

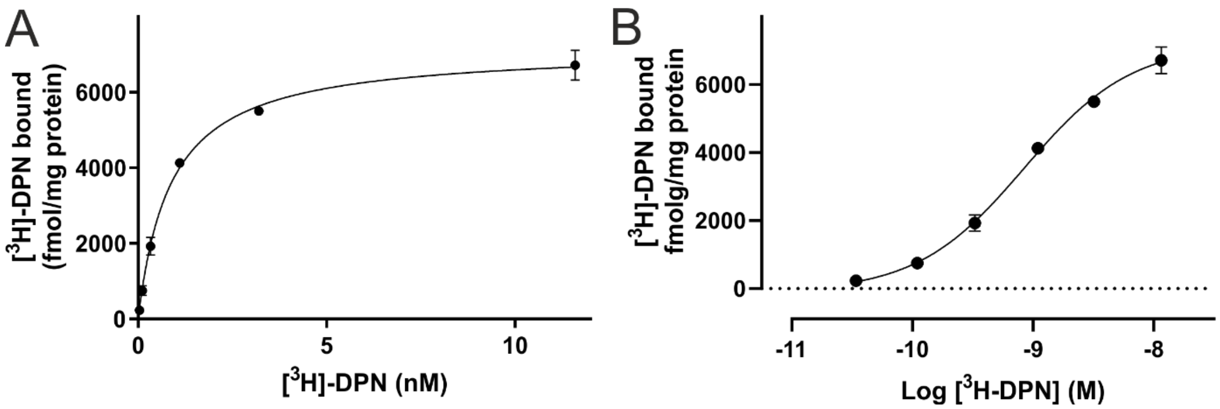

Supporting Information Figure S1. Saturation binding of [^{3}H]-DPN at the mouse KOR. Distinct concentration of [^{3}H]-diprenorphine (^{3}H-DPN) were incubated for 1 h at 37°C in HEK293 cells stably expressing mouse κ-opioid receptor (KOR). Data were fitted to (A) one-site binding model to derive K_d and B_{max} values or (B) with four-parameter logistic Hill equation to determine Hill slope. Data are presented as mean ± SD (n=2).

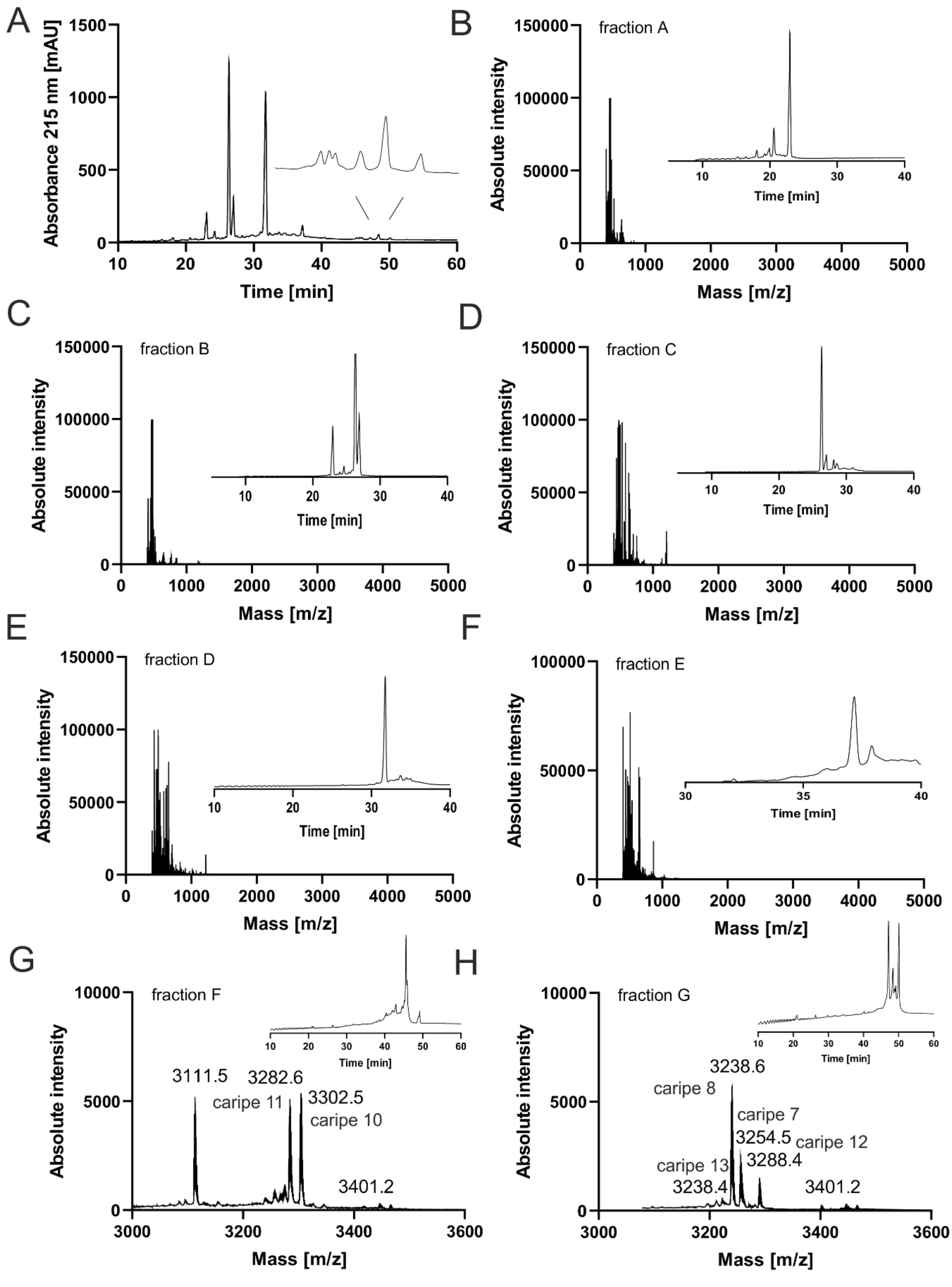

Supporting Information Figure S2. HPLC fractionation of ipecac extract. (A) HPLC profile of ipecac extract. The inset indicates the eluting region of cyclotides in RP-HPLC. (B) – (F) MALDI mass spectra and chromatograms of fractions containing hydrophilic components of ipecac extract. (G) and (H) MALDI mass spectra and chromatograms of fractions enriched with caripe cyclotides. Shown are monoisotopic masses of caripe 7 (3254.5 Da), caripe 8 (3238.6 Da), caripe 10 (3302.5 Da), caripe 11 (3282.6 Da), caripe 12 (3288.4 Da) and caripe 13 (3238.4 Da) as $[M+H]^+$.

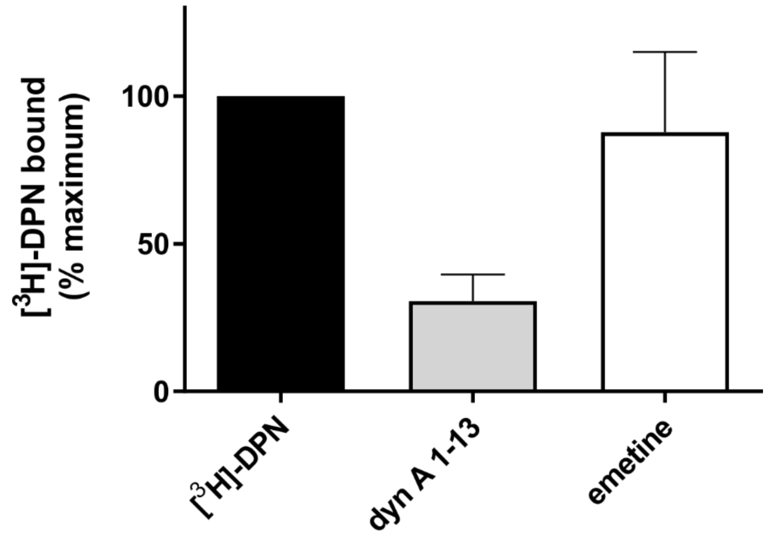

Supporting Information Figure S3. Receptor pharmacology of emetine at the KOR. Displacement binding of 10 µM emetine by 1 nM of tritiated diprenorphine ($[^3H]$-DPN) was tested in HEK293 cells stably expressing the mouse KOR (n=2). Dynorphin (dyn) A_{1-13} was used as a positive control (10 nM).

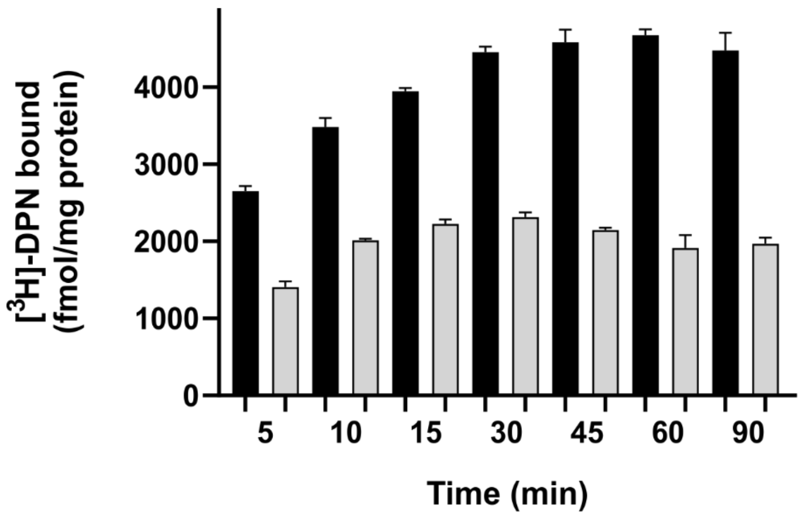

Supporting Information Figure S4. Time course analysis of [^{3}H]-DPN and T20K in the radioligand binding assay at the KOR. [^{3}H]-diprenorphine (^{3}H-DPN, 1 nM) was incubated either alone (black bars) or in combination with 10 µM of T20K (grey bars) over 90 min in HEK293 cells stably expressing mouse κ-opioid receptor (KOR) to examine the duration until equilibrium is reached. Data are shown as mean ± SD of specific binding (n=2).

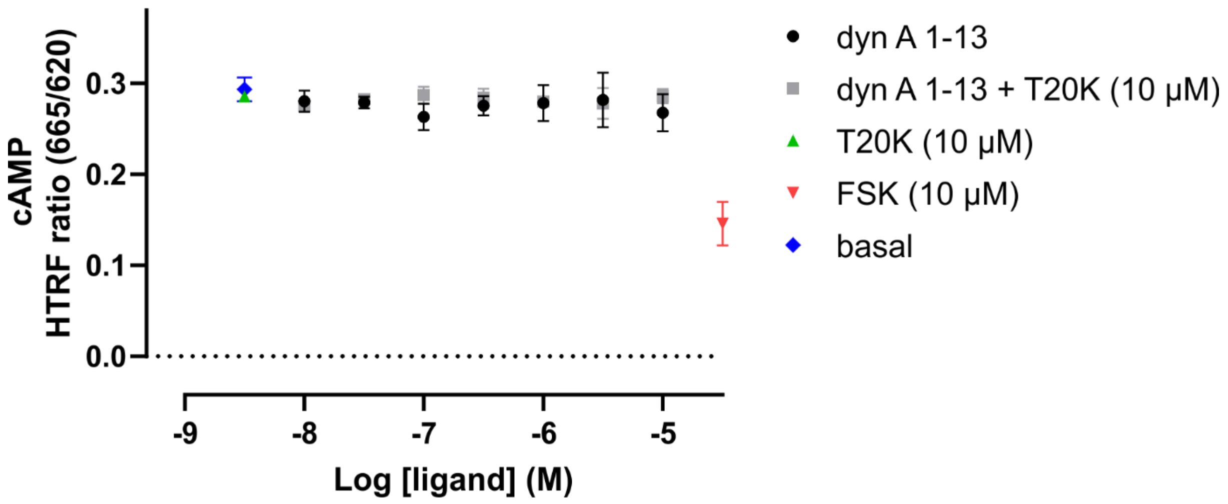

Supporting Information Figure S5. cAMP assay of dyn A_{1-13} without or with T20K in un-transfected HEK293 cells. Varying concentrations of dynorphin (dyn) A_{1-13} were incubated without or with 10 µM of T20K in un-transfected HEK293 cells. 10 µM of forskolin (FSK) were used as positive control. The HTRF ratio signal (665 nM/620 nM) is inversely proportional to endogenously produced cAMP levels. Data are shown as mean ± SD (n=3).

Supporting Information Table S1. Sequences of cysteine-rich peptides identified in plant extracts

Peptide	Sequence	Peptide details	References
caripe 7	CGESCVFIPCTVTALLGCSCKNKVCYRNGIP	cyclotide, cyclic cystine knot, three disulfide bonds (I-IV, II-V, III-VI)	[1]
caripe 8	CGESCVFIPCITAAIGCSCKKKVCYRNGVIP	cyclotide, cyclic cystine knot, three disulfide bonds (I-IV, II-V, III-VI)	[1]
caripe 10	CGESCVFIPCFSTVIGCSCKNKVCYRNGVIP	cyclotide, cyclic cystine knot, three disulfide bonds (I-IV, II-V, III-VI)	[1]
caripe 11	CGESCVFIPCISTVIGCSCKKKVCYRNGVIP	cyclotide, cyclic cystine knot, three disulfide bonds (I-IV, II-V, III-VI)	[1]
caripe 12	CGESCVFIPCFSSVIGCSCKNKVCYRNGVIP	cyclotide, cyclic cystine knot, three disulfide bonds (I-IV, II-V, III-VI)	[1]
caripe 13	CGESCVFIPCFTSVFGCSCKDKVCYRNGIP	cyclotide, cyclic cystine knot, three disulfide bonds (I-IV, II-V, III-VI)	[1]
psypoe 1	CGETCFTTVCNTPGCYCGAYXCTRNGSVI*	cyclotide, cyclic cystine knot, three disulfide bonds (I-IV, II-V, III-VI)	[2]
T20K	CGETCVGGTCNTPGCKCSWPVCTRNGLPV	cyclotide, cyclic cystine knot, three disulfide bonds (I-IV, II-V, III-VI)	[3]
MCTI-III	CPRILKQCKQDSDCPGECICMAHGFCGERG	trypsin inhibitor, inhibitor cystine knot, three disulfide bonds (I-IV, II-V, III-VI)	[4]
bevuTI-I	CTPSGTICSPEAPEQCCSNSCVPHQWLRIFVCA	trypsin inhibitor, six cysteines, cystine connectivity I-IV, II-V, III-VI	[5]
CRP-I	CGESCYLIPCFTPGCYCVSRQCVNKNGEY[#]	cyclotide-like knottin peptide, six cysteines, cystine connectivity not determined	[6]

***X** represents an unidentified amino acid

[#]Peptide was only partially sequenced and examined by homology to known cyclotide sequences, thus representing a putative cyclotide sequence

REFERENCES

1. Fahradpour, M.; Keov, P.; Tognola, C.; Perez-Santamarina, E.; McCormick, P. J.; Ghassempour, A.; Gruber, C. W., Cyclotides isolated from an ipecac root extract antagonize the corticotropin releasing factor type 1 receptor. *Front Pharmacol* **2017,** *8*, 616.
2. Koehbach, J.; Attah, A. F.; Berger, A.; Hellinger, R.; Kutchan, T. M.; Carpenter, E. J.; Rolf, M.; Sonibare, M. A.; Moody, J. O.; Wong, G. K.; Dessein, S.; Greger, H.; Gruber, C. W., Cyclotide discovery in Gentianales revisited--identification and characterization of cyclic cystine-knot peptides and their phylogenetic distribution in Rubiaceae plants. *Biopolymers* **2013,** *100*, 438-452.
3. Thell, K.; Hellinger, R.; Sahin, E.; Michenthaler, P.; Gold-Binder, M.; Haider, T.; Kuttke, M.; Liutkeviciute, Z.; Goransson, U.; Grundemann, C.; Schabbauer, G.; Gruber, C. W., Oral activity of a nature-derived cyclic peptide for the treatment of multiple sclerosis. *Proc Natl Acad Sci U S A* **2016,** *113*, 3960-3965.
4. He, W. J.; Chan, L. Y.; Clark, R. J.; Tang, J.; Zeng, G. Z.; Franco, O. L.; Cantacessi, C.; Craik, D. J.; Daly, N. L.; Tan, N. H., Novel inhibitor cystine knot peptides from Momordica charantia. *PLoS One* **2013,** *8*, e75334.
5. Retzl, B.; Hellinger, R.; Muratspahic, E.; Pinto, M. E. F.; Bolzani, V. S.; Gruber, C. W., Discovery of a beetroot protease inhibitor to identify and classify plant-derived cystine Knot peptides. *J Nat Prod* **2020,** *83*, 3305-3314.
6. Alvarez, C. A.; Barriga, A.; Albericio, F.; Romero, M. S.; Guzman, F., Identification of peptides in flowers of Sambucus nigra with antimicrobial activity against aquaculture pathogens. *Molecules* **2018,** *23*.
